# Disinfection Procedures and Their Effect on the Microorganism Colonization of Dental Impression Materials: A Systematic Review and Meta-Analysis of In Vitro Studies

**DOI:** 10.3390/bioengineering9030123

**Published:** 2022-03-16

**Authors:** Louis Hardan, Rim Bourgi, Carlos Enrique Cuevas-Suárez, Monika Lukomska-Szymanska, Elizabeth Cornejo-Ríos, Vincenzo Tosco, Riccardo Monterubbianesi, Sara Mancino, Ammar Eid, Davide Mancino, Naji Kharouf, Youssef Haikel

**Affiliations:** 1 Department of Restorative Dentistry, School of Dentistry, Saint-Joseph University, Beirut 1107 2180, Lebanon; louis.hardan@usj.edu.lb (L.H.); rim.bourgi@net.usj.edu.lb (R.B.); 2 Dental Materials Laboratory, Academic Area of Dentistry, Autonomous University of Hidalgo State, Circuito Ex Hacienda La Concepción S/N, San Agustín Tlaxiaca 42160, Mexico; elizabeth_cornejo@uaeh.edu.mx; 3 Department of General Dentistry, Medical University of Lodz, 251 Pomorska St., 92-213 Lodz, Poland; monika.lukomska-szymanska@umed.lodz.pl; 4 Department of Clinical Sciences and Stomatology, Polytechnic University of Marche, 60121 Ancona, Italy; v.tosco@pm.univpm.it (V.T.); r.monterubbianesi@univpm.it (R.M.); 5 Faculty of Pharmacy, Strasbourg University, 67400 Strasbourg, France; sara.mancino@etu.unistra.fr; 6 Department of Endodontics, Faculty of Dental Medicine, Damascus University, Damascus 0100, Syria; ammarendo89@gmail.com; 7 Department of Biomaterials and Bioengineering, INSERM UMR_S 1121, Biomaterials and Bioengineering, 67000 Strasbourg, France; endodontiefrancaise@outlook.com (D.M.); youssef.haikel@unistra.fr (Y.H.); 8 Department of Endodontics, Faculty of Dental Medicine, Strasbourg University, 67000 Strasbourg, France; 9 Pôle de Médecine et Chirurgie Bucco-Dentaire, Hôpital Civil, Hôpitaux Universitaire de Strasbourg, 67000 Strasbourg, France

**Keywords:** antibacterial effect, dental impressions, disinfectant agents, disinfection, oral bacteria

## Abstract

Dental impressions are contaminated with potentially pathogenic microorganisms when they come into contact with patient blood, saliva, and plaque. Numerous disinfectants are used; however, no sole disinfectant can be designated as universal for all the impression materials. Thus, the aim of this study is to systemically review the literature to evaluate the effect of the existing disinfection procedures on the bacterial colonization of dental impression materials. This systematic review and meta-analysis was conducted according to the PRISMA statement. PubMed (MEDLINE), Web of Science, Scopus, EMBASE, and SciELO databases were screened up to April 2021. Eligibility criteria included in vitro studies reporting the antibacterial activity of disinfectant solutions in dental impression materials. The meta-analysis was performed using Review Manager (version 5.3.5). A global comparison was performed with the standardized mean difference based on random-effect models at a significance level of α = 0.05. A total of seven studies were included in the meta-analysis. The included studies described the effect of disinfection processes with chlorhexidine gluconate, alcohol, sodium hypochlorite, glutaraldehyde, and hydrogen peroxide in alginate, polyvinyl siloxane, and polyether impression materials. The meta-analyses showed that the use of chlorhexidine, alcohol, glutaraldehyde, and sodium hypochlorite reduced the colony-forming units by a milliliter (CFU/mL) in alginate (*p* < 0.001). On the other hand, glutaraldehyde, sodium hypochlorite, and alcohol reduced the CFU/mL in polyvinyl siloxane (*p* < 0.001). Finally, alcohol and glutaraldehyde reduced the CFU/mL in polyether material (*p* < 0.001). High heterogenicity was observed for the alginate and polyvinyl siloxane materials (I^2^ = 74%; I^2^ = 90%). Based on these in vitro studies, the disinfection of impression materials with several disinfection agents reduces the CFU/mL count.

## 1. Introduction

Dental impressions are certainly contaminated with possibly pathogenic microorganisms when they come into contact with patient blood, saliva, and plaque [1,2]. This could be the source of disease transmitters and cross-infections for dentists, dental assistants, and laboratory technicians [3,4]. Consequently, sanitizing the impressions efficiently before transportation to the laboratory technician ensembles is crucial [5]. Indeed, when the impressions are sterilized, this can avoid the transmission of disease, yet it is not the ideal way, since dimensional changes can occur [6].

Considering that, in some countries, tap water contains halogenated compounds, the Advisory British Dental Association Service recommends the rinsing of impression materials with tap water in daily dental practice; despite this, although some of the microorganisms adhered to the surface of a dental impression could be removed by this procedure, a high percentage still remains [7]. This has been exhibited to lessen the amounts of the bacteria on the surface of the impression presented by nearly 90% [8]. Nevertheless, a noteworthy number of bacteria would persist. More recent suggestions support the use of a disinfecting solution [9]. Knowledge evidently varies about the type, concentration, and immersion times of disinfection protocols, making it difficult to evaluate the most applicable method [10,11].

Numerous disinfectants are used regularly such as sodium hypochlorite, chlorhexidine, alcohol, glutaraldehyde, and hydrogen peroxide [12]. Since no sole disinfectant can be designated as a universal disinfectant for all impression materials, it is fundamental to select an ideal disinfectant agent with superior antimicrobial activity that does not disturb the recorded features, such as surface characteristics or dimensional stability of an impression materials [13,14].

Additionally, many combinations between impression materials and disinfectant could occur by knowing that a large range of branded impression materials (reversible and irreversible hydrocolloids, polyethers, polysulphides, and silicones) and gypsum-based casts existed in the marketplace. A disinfectant possesses a dual purpose: it needs to be an effective antimicrobial agent but produce no adverse effect on the dimensional accuracy of the impression material and resultant gypsum model. The latter is of significance in an attempt to deliver a functional and well-fitting finished appliance. Disagreement happens in the literature as to whether the disinfection procedure produces degradation or distortion of impressions [15,16,17].

The reaction of some specific brands of gypsum products and impression materials to disinfection process is diverse, advising a deficiency of compatibility between a given material and protocol. Hence, individual analysis of impression materials is needed to define the effectiveness of a specific disinfection method in different areas [18].

Accordingly, the aim of this study was to systemically review the literature of the existing disinfection procedures on the bacterial colonization of dental impression materials. The null hypothesis to be tested was that the use of disinfectant agents will not reduce the colony-forming units per milliliter (CFU/mL) adhered to the surface of impression materials used in dentistry.

## 2. Materials and Methods

This systematic review and meta-analysis was reported following the guidelines of the Preferred Reporting Items for Systematic Reviews and Meta-Analyses (PRISMA statement) [19]. The registration protocol was carried out in the Open Science Framework with the registration number 0000-0002-2759-8984. The following PICOS strategy was used: population, impression materials; intervention, use of disinfection materials; control, rinsing with tap water; outcome: antimicrobial activity; and type of study, in vitro studies. The research question was as follows: Does the use of disinfection procedures for impression materials in dental practice reduce the microbial count?

### 2.1. Search Strategy

The literature search was performed by two independent reviewers (E.C.R. and R.B.) up to April 22, 2021. The following databases were screened: PubMed (MEDLINE), Web of Science, Scopus, EMBASE, and SciELO. The search strategy was performed according to the keywords defined in Table 1. All studies were imported into Rayyan QCRI platform [20].

### 2.2. Eligibility Criteria

The title and abstract of each identified article were reviewed by two independent reviewers (E.C.R. and R.B.) to determine if the article should be considered for full-text review according to the following eligibility criteria: (1) in vitro studies reporting the antibacterial activity of disinfectant solutions in dental impression materials; (2) included mean and standard deviation (SD) in CFU/mL; (3) included a control group where tap water was used; and (4) published in the English language. Case reports, case series, pilot studies, expert opinions, conference abstracts, and reviews were excluded. In the case of disagreements at the time of the selection of the studies for the full-text review, they were resolved by discussion and consensus by a third reviewer (C.E.C.-S).

### 2.3. Data Extraction

The Microsoft Office Excel 2019 program (Microsoft Corporation, Redmond, Washington, DC, USA) was used to extract the data of interest from the included manuscripts. These were placed on a standardized form. Two reviewers (L.H. and R.B.), who received training in this software, performed the analysis. The data recovered from each manuscript were author, year, impression material evaluated, disinfection agents used, type of microorganism evaluated, main outcome, and main results.

### 2.4. Quality Assessment

The risk of bias of the selected articles was assessed by two reviewers (R.B. and E.C.R.) according to the parameters of the previous systematic review [21]. The risk of bias of each article was evaluated according to the description of the following parameters: specimen randomization, single-operator protocol implementation, blinding of the operator, the presence of a control group, complete outcome data, and description of the sample size calculation. If the authors reported the parameter, the study received a “YES” for that specific parameter. In case of missing information, the parameter received a “NO”. The risk of bias was classified according to the sum of “YES” answers received: 1 to 2 indicated a high bias, 3 to 4 indicated a medium risk of bias, and 5 to 6 indicated a low risk of bias.

### 2.5. Statistical Analysis

The meta-analyses were performed using Review Manager Software version 5.1 (The Nordic Cochrane Centre, The Cochrane Collaboration, Copenhagen, Denmark). The analyses were carried out using a random-effect model, and pooled-effect estimates were obtained by comparing the standardized mean difference between CFU/mL values obtained when a disinfection agent was used; against a control group when tap water was used. The standardized mean difference was performed since this statistic in meta-analysis is used when all the studies assess the same outcome but measure it in a variety of ways; for this to be appropriate, it must be assumed that between-study variation reflects only differences in measurement scales, such as the different scientific notation used among the studies included. Additionally, for comparison purposes, when a value of 0 was found in the data, this was replaced with “0.1” with a SD of “0.01” for the statistical analysis. The comparisons were made considering the type of impression material and the type of disinfection agent used. A *p*-value < 0.05 was considered statistically significant. Statistical heterogeneity of the treatment effect among studies was assessed using the Cochran Q test and the inconsistency I^2^ test.

## 3. Results

**Figure 1:**
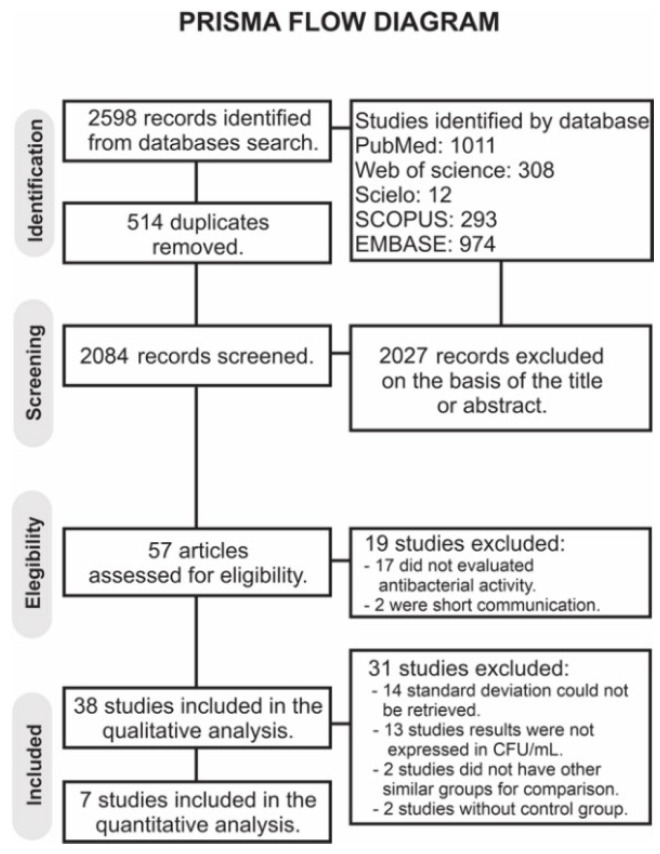
Search flowchart according to the PRISMA Statement.

The search resulted in the retrieval of 2598 records (Figure 1). After removal of duplicates, 2084 articles were screened, and 2027 were excluded based on the title or abstract. A total of 57 full-text articles were assessed for eligibility. Of these, nineteen were not considered for the qualitative analysis: seventeen did not evaluate the antibacterial activity and two were short communications, leaving thirty-eight studies for the qualitative analysis; from these, thirty-one were excluded from the quantitative analysis: in fourteen studies, the SD could not be retrieved, and in another thirteen studies, the results were not expressed in CFU/mL, two studies did not have any control group, and two studies did not have enough comparison groups. Finally, seven studies were considered for the meta-analysis. Table S1 describes the quantitative data extracted from studies included in the meta-analysis.

The characteristics of the studies included in this systematic review are summarized in Table 2. Several disinfection agents were identified for the present review, including chlorhexidine, alcohol, sodium hypochlorite, glutaraldehyde, and hydrogen peroxide. Most of the studies included in this review evaluated the antibacterial activity to alginate and polyvinyl siloxane impressions, only two studies evaluated the effect of disinfection on polyether, while only one tested on condensation silicone. Utmost of the studies reported the effect of disinfection agents on CFU/mL, while a few reported inhibition halos.

**Figure 2:**
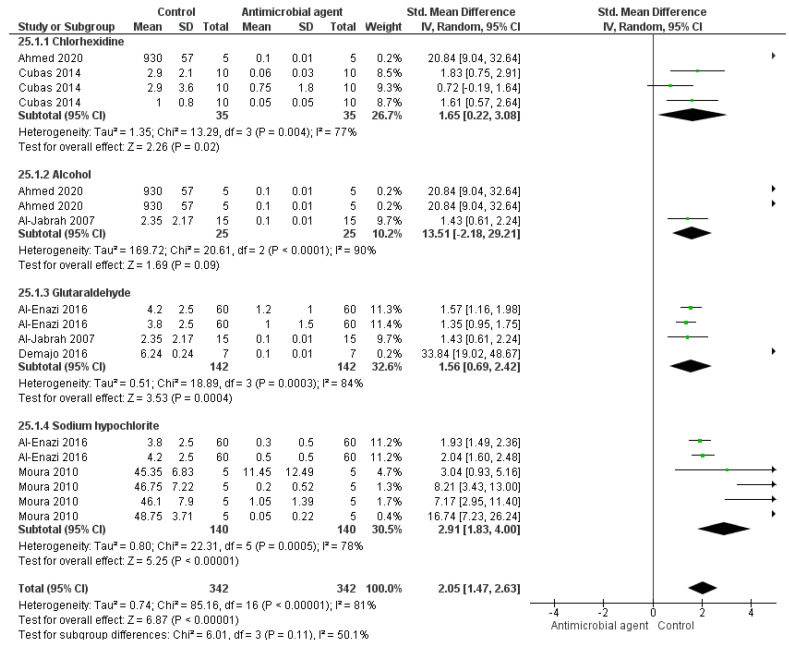
Forest plot of the analysis of CFU/mL count in alginate after disinfection.

**Figure 3:**
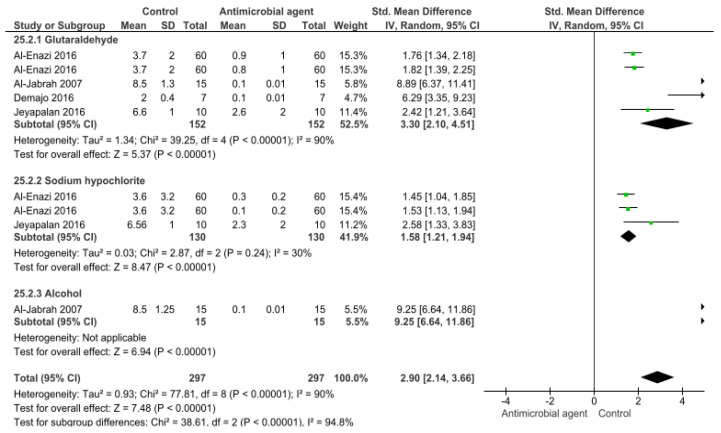
Forest plot of the analysis of CFU/mL count in polyvinyl siloxane after disinfection.

**Figure 4:**
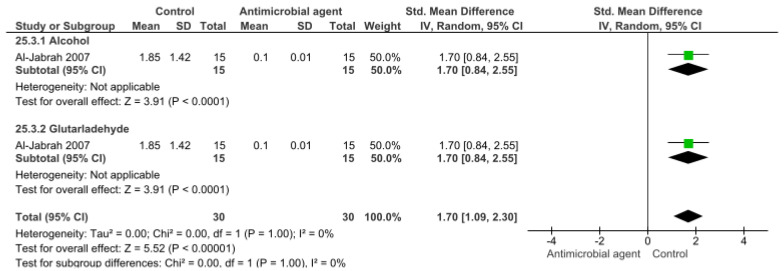
Forest plot of the analysis of CFU/mL count in polyether after disinfection.

Figure 2, Figure 3 and Figure 4 show the result from the meta-analyses. With regards to alginate, the use of disinfection agents such as chlorhexidine, alcohol, glutaraldehyde, and sodium hypochlorite significantly reduced the CFU/mL count (*p* < 0.001). It is worth mentioning that a high heterogenicity was observed (I^2^ = 74%) (Figure 2).

Figure 3 shows the effect of different disinfection agents on polyvinyl siloxane material. According to the meta-analysis, all the disinfection agents tested significantly reduced the CFU/mL count (*p* < 0.001). Again, a high heterogenicity was observed in the comparisons (90%).

Finally, Figure 4 shows the effect of different disinfection agents on polyether impression material. According to the meta-analysis, both alcohol and glutaraldehyde significantly reduced the CFU/mL count (*p* < 0.001). As only one study was included in this analysis, a 0% heterogenicity was found.

The risk of bias analysis was shown that most of the studies were categorized with high and medium risk of bias (Table 3). Utmost of the manuscripts examined failed to report the single operator, operator blinded, and sample size calculation factors.

## 4. Discussion

This systematic review and meta-analysis was directed towards testing the effect of disinfection agents on the bacterial colonization of different impression materials. This review focused on the study of the CFU/mL measure, since this is the most common measure used to determine the antibacterial activity. To the best of the authors knowledge, this is the first approach to prove that the application of disinfectant agents is effective to reduce the count of some oral pathogens on the surface of alginate, polyvinyl siloxane, and polyether impression materials and that this procedure can certainly reduce the possibility of cross-contamination. Accordingly, the hypothesis tested in this study was rejected.

Normally, chemical disinfectant agents were generally used in dental exercise because of their easy application. For the alginate materials, the use of disinfection agents such as chlorhexidine, alcohol, glutaraldehyde, and sodium hypochlorite significantly reduced the CFU/mL count (*p* < 0.001). Irreversible hydrocolloids, the frequent material used in dentistry, tend to absorb both blood and saliva [23]. Thus, research was focused on a solution to inhibit the colonization of microbe on the surface of these materials [11].

Collected data were established on the CFU in a media culture. These were recorded by using a colony counter, and the counts were expressed by a standard technique of estimating microbial colony count known as the CFU count. The bacteriological examination evidently exhibited that the CFU recorded after disinfection were fewer than before disinfection [23], thus making the disinfection process an important issue to solve after taking an impression in the dental world.

It is highlighted in a previous study [58] that the use of tap water on the surface of alginate impression failed to kill *Streptococcus Mutans* and *Lactobacilli*; however, by using chlorhexidine, a positive antimicrobial activity has been shown [59]. This could be possible by the binding between the positive site of chlorhexidine and negative sites of the bacterial cell, which resulted in interference with osmosis and escapes the constituents that lead to cell death [22]. In addition, alcohol was able to kill all the detected bacteria in this study by inactivating the growth of the bacteria on the alginate impression, and this was deemed probable by alkylating the amino and sulf hydral groups of bacterial proteins [60,61,62]. Further, for the other disinfectants, it was demonstrated that by using 2% glutaraldehyde solution or 1% sodium hypochlorite, gram-positive organisms will be modified by reducing their growth [11]. Indeed, this effect was most noticeable for 1% sodium hypochlorite, as described in this research.

A previous study denoted that after immersion in sterile water for 10 min, for some of impression materials, including alginate impression, the number of microorganisms counted was diminished, though alginate material still retained some of these microorganisms in comparison to other materials [24]. The physical nature of alginate impression could affect the capacity of disinfectants for doing their biocidal activity. In the oral environment, microorganisms might become integrated into the gelling impression material since the presence of oral fluids or saliva [29]. The set-up of these microorganisms in the alginate material restricted the efficacy of the water rinse, and the alginate gel assembly could hinder the penetration of the disinfectant [29,63]. Thus, this idea explained the results of this study as tap water did not reduce the microorganism counts in comparison to the other disinfectant solution. Overall, for alginate impressions, the use of disinfectant agents would be of great interest, and the efficacy of the disinfection ranged between 92% and 99.97% in all the situations [23].

According to the meta-analysis, all the disinfection agents significantly reduced the CFU/mL count (*p* < 0.001) on polyvinyl siloxane material. Among the numerous available impression materials in prosthodontics, this material was considered the material of choice, due to their fine detail reproduction, excellent physical properties, remarkable dimensional stability, good acceptance by the patient, and elastic recovery feature [64,65,66]. In addition, these materials were tasteless and odorless [46]. As stated above in alginate impression, using 2% glutaraldehyde solution or 1% sodium hypochlorite could be recommended also for disinfecting the polyvinyl siloxane impression [67,68]. In this manner, it is advisable to immerse these kinds of impressions in these solutions rather than spraying, as successful finding was observed in many previous studies, without harming the physical properties [15,18,31,69]. Seemingly, by putting the polyvinyl siloxane material in an alcohol-based disinfectant solution for a contact time of 15 min, a media free of microorganisms could be observed [24]. Accordingly, this can support the finding obtained in this study, as any kind of disinfectant solution tested showed promising results with polyvinyl siloxane impression material.

The present analysis noted that both alcohol and glutaraldehyde significantly reduced the CFU/mL count (*p* < 0.001) of polyether impression material. This could be explained by the fact that the contaminating bacteria could be reduced by 85% when soaking this kind of impression material in sterile water for 15 min; in addition, as found with polyvinyl siloxane impression, the use of alcohol-based solution produced effective disinfection of the polyether impression [24]. With regards to glutaraldehyde, the antimicrobial activity of this compound depends on the duration of dilution and its concentration. This could be elucidated by the fact that the biocidal activity of glutaraldehyde results from alkylation of sulfhydryl, hydroxyl, carboxyl, and amino groups of microorganisms, which alters RNA, DNA, and protein synthesis [70]. This conclusion seems to support the results in this meta-analysis.

From this review, various disinfectant agents were used to show the importance of reducing microorganisms on the surface of the impression materials used in dentistry. The results should be considered with caution since other brands of impression materials were available in the dental market and not included. In addition, there is the opportunity for slight changes in chemistry of these materials, causing significantly different reactions. Additionally, most of the studies included were classified as having high or medium risk of bias, and, therefore, better experimental designs should be conducted in order to obtain a higher degree of evidence. One of the limitations of this review relies on the fact that it is only focusing on the antibacterial efficacy of the application of a disinfectant on the surface of a dental impression; however, other variables should be taken into account, such as the effect of this procedure on the accuracy, precision, and surface quality of the resulting working models, especially when dental impressions are disinfected both in the dental office and in the dental laboratory. This led to controversy as to whether the disinfection process causes degradation or distortion of dental impressions and to what extent. Therefore, studying the effect of these disinfectants on the dimensional stability of the impression materials should be considered in further research. Additionally, viruses could be considered in future investigation by having the required equipment since their manipulation was considered dangerous for some researchers. Moreover, clinical studies were needed since testing of the efficacy of disinfectants from different patients derived impressions was scarce, knowing the differences in oral flora composition of individual person.

## 5. Conclusions

Based on in vitro studies, disinfection of alginate with chlorhexidine, alcohol, glutaraldehyde, and sodium hypochlorite reduced the CFU/mL count on the surface of alginate impressions. This trend was observed when polyvinyl siloxane impressions were disinfected with glutaraldehyde, sodium hypochlorite, and alcohol and when polyether was immersed in alcohol or glutaraldehyde. Therefore, these substances could be employed to reduce cross-contamination in the dental office.

## Figures and Tables

**Table 1:** Keywords used in search strategy.

Search Strategy	
# 1	Dental models OR Dental impressions OR Irreversible hydrocolloid OR Alginate impressions OR Silicone impression OR Primary impression OR Polyvinyl siloxane
# 2	Disinfection OR Sodium hypochlorite OR Disinfection techniques OR Sterilization OR Chemical disinfection OR Disinfection protocol OR Immersion disinfection OR Cross contamination OR Ultraviolet disinfection OR Microbial activity OR Disinfectant solutions OR Autoclave OR Disinfectant agents
# 3	#1 and #2

**Table 2:** Characteristics of the included studies.

Study	Impression Material	Disinfection Agent	Type of Microorganism	Main Outcome	Main Results
Ahmed 2020 [22]	Alginate	Chlorhexidine Desident CaviCide Alcohol	*Bacteria* *Fungi*	Colony-forming units	Disinfectants killed the bacteria completely.
Al-Enazi 2016 [23]	Polyvinyl siloxane Alginate	Sodium hypochlorite Glutaraldehyde	*Streptococcus* *diphtheroid* *Neisseria*	Colony-forming units	Use of 1% sodium hypochlorite yielded better results than did 2% glutaraldehyde.
Al-Jabrah 2007 [24]	Alginate Polyether Polyvinyl siloxane	Dimenol Perform-ID^®^ MD520^®^ Haz-tabs^®^	Does not specify	Colony-forming units	All four disinfectant solutions tested produced effective disinfection of the impression materials investigated.
Alwahab 2012 [25]	Alginate	Chlorhexidine digluconate	*Pseudomonas aeruginosa* *Staphylococcus aureus* *Candida albicans*	Inhibition halos	The least antibacterial activity of chlorhexidine digluconate was observed against *Pseudomonas aeruginosa.*
Azevedo 2019 [26]	Polyvinyl siloxane	Hydrogen peroxide MD520^®^ Sodium hypochlorite	Does not specify	Colony-forming units	All disinfectants tested showed high antimicrobial efficiency.
Bal 2007 [27]	Polyvinyl siloxane Polyether	Sodium hypochlorite Gludex spray Mikrozid spray	*Staphylococcus aureus* *Enterococcus faecalis*	Colony-forming units	The disinfectant spray was less effective than sodium hypochlorite or Gludex.
Benakatti 2017 [28]	Alginate	Chlorhexidine Gluconate solution	*Staphylococcus aureus*	Inhibition halos	This disinfection method was effective in the elimination of *S. aureus.*
Beyerle 1994 [29]	Alginate	Sodium Hypochlorite	*Bacillis subtilis* *Mycobacleriuiii bovis*	Colony-forming units	One-minute exposure resulted in very inconsistent killing in all instances.
Brauner 1990 [30]	Alginate	Blueprint asept^®^	*Streptococcus mutans* *Streptococcus sanguis* *Streptococcus aureus* *Streptococcus pyogenes* *Staphylococcus aureus* *Actinomyces odontolyticus* *Escherichia coli* *Klebsiella pneumoniae* *Proteus mirabilis* *Enterobacter aerogenes* *Pseudomonas aeruginosa*	Inhibition halos	Due to its bactericidal effect, Blueprint asept^®^ can be recommended.
Bustos 2010 [31]	Alginate Condensation silicone Alginate	Sodium Hypochlorite Glutaraldehyde	*Gram (+) and (-) coccus and Gram (-) bacillus* *Candida*	Colony-forming units	Alginate and silicone impressions can successfully be disinfected if they are immersed in either 0.5% NaOCl solution or 2% glutaraldehyde for 5 min.
Choudhury 2018 [32]	Alginate	Sodium Hypochlorite Epimax^®^	*Staphylococcus aureus* *Candida albicans* *Pseudomonas aeruginosa*	Colony-forming units	Both Epimax and 0.525% sodium hypochlorite can disinfect the alginate impression material against *Candida albicans*, *Pseudomonas aeruginosa*, and *Staphylococcus aureus.*
Cserna 1994 [33]	Alginate	Chlorhexidine Quaternary ammonium salt	*Lactobacillus* *Streptococcus mutans*	Inhibition halos	Antimicrobial alginates are more effective than nonantimicrobial alginates in reducing the surface growth of the oral bacteria *Lactobacillus* and *Streptococcus mutans.*
Cubas 2014 [34]	Alginate	Chlorhexidine	*Streptococci* *Candida*	Colony-forming units	Chlorhexidine as a water substitute during impression taking offers decreased microbial contamination with no negative alterations of the resulting casts, thus providing an easy method for controlling cross-infection.
Demajo 2016 [35]	Alginate Polyvinyl siloxane	MD 520^®^ Minuten^®^	Does not specify	Colony-forming units	Glutaraldehyde is more effective than alcohol-based chemical disinfectants.
Doddamani 2011 [36]	Alginate	Povidone Iodine Sodium Hypochlorite Glutaraldehyde Distilled Water	*Staphylococcus aureus* *Bacillus subtilis* *Streptococcus viridans*	Colony-forming units	Disinfectants work equally well on an irreversible hydrocolloid impression material.
Estafanous 2012 [37]	Polyvinyl siloxane Polyether	EcoTru [EnviroSystems] ProSpray [Certol] Sodium hypochlorite	*Pseudomonas aeruginosa* *Salmonella choleraesius* *Staphylococcus aureus*	Colony-forming units	Disinfectants investigated in this study will effectively disinfect Polyvinyl siloxane and polyether elastomeric impression materials.
Flanagan 1998 [38]	Alginate	Single quaternary ammonium compound Chlorhexidine Dual quaternary ammonium compound	*Gram-positive cocci* *Gram-negative bacilli* *yeast*	Colony-forming units	The alginate with chlorhexidine killed all the gram-negative bacilli and the majority (95–99%) of the gram-positive cocci and yeast.
Gerhardt 1991 [39]	Alginate	Sodium hypochlorite	*Staphylococcus aureus* *Pseudomonas aeruginosa* *Bacillus subtitis*	Inhibition halos	The results indicated that chlorine disinfecting solutions of sufficient concentration can be retained for periods up to 1 week and still maintain their effectiveness.
Ginjupalli 2016 [40]	Alginate	Silver nanoparticles	*E. coli* *S. aureus* *C. albicans*	Inhibition halos	The particles imparted significant antimicrobial activity to the alginate impression materials tested.
Goel, 2014 [41]	Alginate	Sodium hypochlorite Microwave irradiation	*Staphylococcus aureus* *Pseudomonas aeruginosa*	Colony-forming units	The results suggested that the microwave irradiated Kala stone casts proved to be a better disinfection method when compared with 0.07% sodium hypochlorite chemically disinfected incorporated cast.
Hiramine 2021 [42]	Alginate	Sodium dichloroisocyanurate NaClO	*Streptococcus mutans* *Escherichia coli* *Staphylococcus aureus* *Candida albicans* *Dental plaque bacteria*	Colony-forming units	The number of oral bacteria adhering to the surfaces of impressions markedly decreased following a 10 min immersion in the 1000 ppm sodium dichloroisocyanurate solution.
Ishida 1991 [43]	Alginate Condensation silicone	UV light	*Candida albicans* *C. glabrota* *C. tropicalis* *C. parupsilosis* *C. krusei* *C. guilliermondi*	Colony-forming units	UV light is effective in disinfecting impression materials that are contaminated with candida organisms.
Ismail 2016 [44]	Alginate	Povidone iodine powder	*Streptococcus mutans and Staphylococcus aureus*	Inhibition halos	Modified alginate impression material with 15 weight % povidone-iodine powered gives the material self-disinfected properties
Ivanovski 1995 [45]	Alginate	Sterile Water Chlorhexidine Glutaraldehyde Povidone-iodine Sodium hypochlorite with sodium chloride	*Escherichia coli* *Staphylococcus aureus* *Enterobacter cloacae* *Pseudomonas aeruginosa* *Klebsiella pneumoniae* *Actinobacter calcoaceticus* *Bacillus subtilis* *Mycobacterium phlei* *Candida albicans.*	Colony-forming units	When glutaraldehyde was used, all the microorganisms tested were killed after 1 h. Chlorhexidine was ineffective against most microorganisms.
Jennings 1991 [3]	Polysulfide rubber Alginate Polyvinyl siloxane	Chlorhexidine gluconate	*C albicans* *P. aeruginosa*	Colony-forming units	Chlorhexidine gluconate (0.2%) was found to be less effective than either glutaraldehyde (2%) or sodium hypochlorite (0.0125%).
Jeyapalan 2018 [46]	Polyvinyl siloxane	Electrolyzed oxidizing water Glutaraldehyde Sodium hypochlorite	*Streptococci* *Staphylococci* *Pseudomonas* *Candida* *Proteus* *Klebsiella* *E. coli*	Colony-forming units	All three chemical disinfectants employed in this study showed acceptable mean log reduction values and kill rate % for antimicrobial efficacy.
Mathew 2017 [47]	Polyvinyl siloxane	Radio frequency glow discharge	*Gram-negative bacilli* *Gram-positive cocci* *Escherichia coli* *Staphylococcus aureus*	Inhibition halos	Ratio glow discharge is a very rapid and handy device, which can disinfect saliva contaminated elastomeric impression material surfaces.
McNeill 1992 [48]	Alginate	Glutaraldehyde Hypochlorite solution chlorine Hygojet system	*Streptococcus sanguis* *poliovirus*	Colony-forming units	Washing the impression for 15 s followed by immersion in 2% glutaraldehyde for 20.0 min or a hypochlorite solution for 7.5 min effectively disinfected the impression.
Moura 2010 [49]	Alginate	Sodium hypochlorite	*Does not specify*	Colony-forming units	5.25% sodium hypochlorite can be used with antimicrobial efficacy, using the humidifier box and nebulizer box methods, and 2.5% sodium hypochlorite was not effective in the nebulizer box method.
Nascimento 2015 [50]	Alginate	Sodium hypochlorite Chlorhexidine	*S. mutans* *S. sanguis* *E. faecalis*	Colony-forming units	4% chlorhexidine was the most suitable disinfectant.
Rweyendela 2009 [13]	Alginate	Chlorinated compounds: Aseptrol Presept	*Candida albicans* *Staphylococcus aureus* *Pseudomonas aeruginosa* *Streptococcus mutans* *Bacillus subtilis spores*	Colony-forming units	The compounds effectively disinfected the alginate in the presence of organic material, but Aseptrol did so after an immersion time of only 1.5 min.
Samra 2010 [51]	Alginate Polyvinyl siloxane	Glutaraldehyde Sodium hypochlorite Ultraviolet chamber	*Streptococcus viridans* *Diphtheroids* *Streptococcus pneumoniae* *Candida albicans* *Pseudomonas aeruginosa* *Staphylococcus albus*	Colony-forming units	All the disinfection systems were effective in reducing the microbial load with the ultraviolet chamber as the most effective.
Savabi 2018 [52]	Alginate	Ozonated water	*Pseudomonas aeruginosa* *Staphylococcus aureus* *Candida albicans*	Colony-forming units	Immersion of alginate impression material in ozonated water for 10 min will not lead to complete disinfection but decreases the microorganisms to a level that can prevent infection transmission.
Schwartz 1996 [53]	Alginate	Sodium hypochlorite	*Staphylococcus aureus* *Salmonella choleraesuis* *Pseudomonas aeruginosa* *Mycobacterium bovis* *Bacillus subtilis*	Colony-forming units	It was found that a 10 min immersion in solutions reduced to pH 7 to 11 consistently produced a 4-log (99.99%) or greater reduction in viable organisms.
Singla 2018 [54]	Polyether	Disinfectant spray Deconex	*Escherichia coli* *Staphylococcus aureus* *Pseudomonas aeruginosa* *Candida albicans*	Colony-forming units	The disinfectant used was effective.
Tanaka 1994 [55]	Alginate	Chlorhexidine	*Streptococcus mitis* *Actinomyces naeslundii* *Staphylococcus aureus* *Veillonella parvula* *Porphyromonas gingivalis* *Candida albicans*	Colony-forming units	The use of an impression material supplemented with 1% chlorhexidine, such as Coe Hydrophilic Gel, may protect clinical staff and dental technicians from at least some bacterial infections associated with impression procedures.
Trivedi 2019 [56]	Alginate	Aloe Vera	*Staphylococcus aureus* *Pseudomonas aeruginosa* *Candida albicans*	Colony-forming units	The effectiveness of aloe vera as a disinfectant was demonstrated.
Zhang 2017 [57]	Elastomer impression material	Glutaraldehyde Ultraviolet radiation	*Human Immunodeficiency Virus* *Hepatitis B virus*	Colony-forming units	Combined use of ultraviolet radiation and 2% glutaraldehyde immersion can eliminate both *Human Immunodeficiency Virus* and *Hepatitis B virus.*

**Table 3:** The results of the risk of bias assessment.

Study	Specimen Randomization	Single Operator	Operator Blinded	Control Group	Complete Outcome Data	Sample Size Calculation	Risk of Bias
Ahmed 2020 [22]	NO	NO	NO	YES	NO	NO	High
Al-Enazi 2016 [23]	YES	NO	NO	YES	YES	NO	Medium
Al-Jabrah 2007 [24]	YES	NO	NO	YES	YES	NO	Medium
Alwahab 2012 [25]	NO	NO	NO	YES	YES	NO	High
Azevedo 2019 [26]	YES	NO	NO	YES	NO	NO	High
Bal 2007 [27]	NO	NO	NO	YES	NO	NO	High
Benakatti 2017 [28]	NO	YES	NO	YES	YES	NO	Medium
Beyerle 1994 [29]	NO	NO	NO	YES	NO	NO	High
Brauner 1990 [30]	YES	NO	NO	YES	NO	NO	High
Bustos 2010 [31]	YES	NO	NO	YES	YES	NO	Medium
Choudhury 2018 [32]	NO	NO	NO	YES	NO	NO	High
Cserna 1994 [33]	NO	NO	NO	YES	YES	NO	High
Cubas 2014 [34]	YES	NO	YES	YES	YES	YES	Low
Demajo 2016 [35]	NO	NO	NO	YES	YES	NO	High
Doddamani 2011 [36]	NO	NO	NO	YES	NO	NO	High
Estafanous 2012 [37]	NO	NO	NO	YES	NO	NO	High
Flanagan 1998 [38]	NO	NO	NO	YES	YES	NO	High
Gerhardt 1991 [39]	NO	NO	NO	YES	NO	NO	High
Ginjupalli 2016 [40]	NO	YES	NO	YES	YES	NO	Medium
Goel 2014 [41]	NO	NO	NO	YES	YES	NO	High
Hiramine 2021 [42]	NO	NO	NO	YES	YES	NO	High
Ishida 1991 [43]	NO	NO	NO	YES	YES	NO	High
Ismail 2016 [44]	NO	NO	NO	YES	NO	NO	High
Ivanovski 1995 [45]	NO	NO	NO	YES	YES	NO	High
Jennings 1991 [3]	YES	NO	NO	YES	YES	NO	Medium
Jeyapalan 2018 [46]	YES	NO	NO	YES	YES	NO	Medium
Mathew 2017 [47]	NO	NO	NO	YES	NO	YES	High
McNeill 1992 [48]	NO	NO	NO	YES	NO	NO	High
Moura 2010 [49]	YES	NO	NO	YES	YES	NO	Medium
Nascimento 2015 [50]	NO	NO	NO	YES	YES	NO	High
Rweyendela 2009 [13]	NO	NO	NO	YES	YES	NO	High
Samra 2010 [51]	NO	NO	NO	YES	NO	NO	High
Savabi 2018 [52]	NO	NO	NO	YES	YES	NO	High
Schwartz 1996 [53]	NO	NO	NO	YES	YES	NO	High
Singla 2018 [54]	NO	NO	NO	YES	NO	NO	High
Tanaka 1994 [55]	NO	NO	NO	YES	NO	NO	High
Trivedi 2019 [56]	NO	NO	NO	YES	YES	YES	Medium
Zhang 2017 [57]	YES	NO	NO	YES	NO	NO	High

## Data Availability

The data presented in this study are available in the article.

## Supplementary Materials

The following supporting information can be downloaded at: https://www.mdpi.com/article/10.3390/bioengineering9030123/s1, Table S1: Quantitative data extracted from studies included in the meta-analysis.
